# MicroRNA–Gene Networks Distinguish Hormone Receptor Status in HER2-Low Breast Cancer: An Integrative Transcriptomic Analysis

**DOI:** 10.3390/genes17030305

**Published:** 2026-03-03

**Authors:** Eduarda Carvalho, Andreia Brandão, Fernando Schmitt, Nuno Vale

**Affiliations:** 1 PerMed Research Group, RISE-Health, Faculty of Medicine, University of Porto, Alameda Professor Hernâni Monteiro, 4200-319 Porto, Portugal; ecferreira@med.up.pt (E.C.); fschmitt@med.up.pt (F.S.); 2 RISE-Health, Department of Pathology, Faculty of Medicine, University of Porto, Alameda Professor Hernâni Monteiro, 4200-319 Porto, Portugal; 3 Cancer Genetics Group, IPO Porto Research Center (CI-IPOP)/RISE@CI-IPOP (Health Research Network), Portuguese Oncology Institute of Porto (IPO Porto), Porto Comprehensive Cancer Center, 4200-072 Porto, Portugal; andreia.aguiar.brandao@ipoporto.min-saude.pt; 4 RISE-Health, Department of Community Medicine, Health Information and Decision (MEDCIDS), Faculty of Medicine, University of Porto, Rua Doutor Plácido da Costa, 4200-450 Porto, Portugal; 5 Laboratory of Personalized Medicine, Department of Community Medicine, Health Information and Decision (MEDCIDS), Faculty of Medicine, University of Porto, Rua Doutor Plácido da Costa, 4200-450 Porto, Portugal

**Keywords:** HER2-low, breast cancer, hormone receptor, HR-positive, HR-negative, TCGA-BRCA, microRNAs, genes

## Abstract

**Background:** HER2-low breast cancer is a biologically heterogeneous subgroup in which hormone receptor (HR) expression critically shapes prognosis and treatment, but the underlying regulatory mechanisms remain unclear. MicroRNAs (miRNAs) are key post-transcriptional regulators of gene expression and may contribute to HR heterogeneity. This study aimed to identify deregulated miRNAs and associated gene networks distinguishing HER2-low/HR-positive from HER2-low/HR-negative tumors, elucidating the molecular mechanisms underlying this divergence. **Methods:** Differential expression analyses of miRNAs and genes were performed using Wilcoxon tests and DESeq2 (|log_2_FC| > 1; FDR-adjusted *p*-value < 0.05). Survival analyses were conducted using Cox proportional hazards models to evaluate the individual miRNAs and miRNA signature. Functional enrichment analyses, including GO, KEGG and Reactome pathways, were performed. Correlation analysis and the miRNA target prediction were integrated to identify regulatory interactions. **Results:** Comparisons between HER2-low/HR-positive and HER2-low/HR-negative tumors identified 165 significantly deregulated miRNAs and 170 strongly deregulated genes. Intersection analysis highlighted miR-9-5p, miR-532-5p and miR-576-5p as specifically associated with HR-negative status. Survival analyses showed non-significant trends for the overall survival and progression-free interval. Functional enrichment analysis revealed hormone-related pathways in HR-positive tumors and immune, inflammatory and proliferative pathways in HR-negative tumors. Integrative correlation and target prediction analyses identified two miRNA–mRNA regulatory axes, miR-576-5p/TGFBI and miR-9-5p/POU2F2. **Conclusions:** Our study demonstrated that HER2-low breast cancer exhibits distinct miRNA and gene expression profiles, which highlight different transcriptomic profiles according to HR status for the first time. Specific miRNA–gene networks may drive transcriptional heterogeneity, serving as potential biomarkers for stratification and as therapeutic targets. These findings provide insight into the molecular basis of HER2-low tumor diversity and support future development of HR-directed therapeutic strategies.

## 1. Introduction

Breast cancer continues to be the most commonly diagnosed cancer worldwide and the primary cause of cancer-associated mortality among women [1]. The molecular classification of breast tumors relies primarily on hormone receptor (HR) expression and human epidermal growth factor receptor 2 (HER2) status, giving rise to luminal A, luminal B, HER2-enriched and triple-negative breast cancer (TNBC) [2,3]. Within this framework, HER2-enriched breast cancer has traditionally been categorized dichotomously as HER2-positive or HER2-negative [4], but the recent recognition of HER2-low breast cancer prompted the ASCO/CAP guidelines for HER2 testing to reconsider this binary model, defining three categories: HER2-positive (immunohistochemistry (IHC) score 3+ or IHC 2+ with positive in situ hybridization (ISH)), HER2-low (IHC 1+ or IHC 2+ with ISH negative) and HER2-negative (IHC 0) [5].

The clinical relevance of the HER2-low category is largely attributable to the demonstrated efficacy of the novel anti-HER2 targeted antibody–drug conjugate (ADC), trastuzumab deruxtecan (T-DXd), in this subgroup in the DESTINY-Breast04 trial [6]. This discovery prompted updates on the guidelines for HER2 testing and improved clinical outcomes for patients that were previously treated for HER2-negative tumors. Despite this advancement, there is no substantial evidence to classify HER2-low as a biological separate entity, rather than as a subset of HER2-negative tumors, remaining a matter of ongoing investigation [7,8,9]. HER2-low breast cancer is a heterogeneous group of tumors driven by HR expression, with the majority being HR-positive and a smaller fraction lacking estrogen receptor (ER) and progesterone receptor (PR) expressions and thus being classified as HR-negative [10,11,12]. However, the biological mechanisms driving this divergence in HR expression within HER2-low tumors remain poorly understood.

MicroRNAs (miRNAs), small non-coding RNAs, constitute a major regulatory layer capable of regulating gene expression through the interaction with the 3′-untranslated regions (UTR) of mRNA targets [13,14]. In addition, these molecules exert a broad biological effect, influencing cell survival, differentiation, proliferation, metabolism, metastasis and apoptosis [15]. Several miRNAs have been reported to directly or indirectly regulate the expression of ESR1, PGR and ErbB2 genes, which lead to the synthesis of ERs, PRs and HER2, respectively. For instance, the upregulation of Twist enhances miR-22 expression, leading to the downregulation of ERα [16]. Consistently, another study demonstrated that increased miR-22 expression reduces ESR1 levels [17]. Progesterone has been reported to indirectly suppress ESR1 expression by downregulating DSCAM-AS1, which in turn elevates miR-130a levels and targets ESR1 [18], while estrogen stimulation increases the expression of miR-26a and miR-181, resulting in downregulation of PGR [19]. Moreover, miR-125a-3p upregulation enhances ErbB2 expression in HER2-positive and TNBC cell populations [20], whereas increased miR-489 expression reduces ErbB2 gene levels [21]. Additionally, aberrant miRNA expression contributes to cancer, with oncogenic miRNAs being upregulated and tumor suppressive miRNAs downregulated, which is associated with therapy resistance [22,23] and shifts in tumor phenotype [24] across the spectrum of breast cancer, resulting in more aggressive tumors.

Despite the extensive research on the role of miRNAs in breast cancer, the specific miRNA landscape of HER2-low tumors has not been comprehensively characterized. Exploring their capacity as regulators of multiple gene expression may provide a deeper insight into the divergence of HR status observed within the HER2-low subgroup. Therefore, this study aims to elucidate the mechanisms underlying the heterogeneity of HER2-low tumors by identifying deregulated miRNAs and associated gene networks capable of distinguishing HER2-low/HR-positive from HER2-low/HR-negative tumors. Understanding these pathways represents a step toward the potential classification of HER2-low as a distinct clinical entity, may improve prognostic accuracy and can pave the way for the development of novel patient-centered therapeutic strategies.

## 2. Materials and Methods

### 2.1. Data Acquisition

Clinical data of breast cancer patients and respective miRNA and gene expression data were obtained from The Cancer Genome Atlas (TCGA) Breast Cancer (BRCA) dataset from UCSC Xena Browser “https://xenabrowser.net/datapages/ (accessed on June 2025)”. A total of 597 primary tumor (TP) samples and 90 normal adjacent tissue (NAT) samples were included. MiRNA expression data were derived from miRNA sequencing count data, normalized to reads per million (RPM), and log_2_-transformed [log_2_(RPM + 1)]. Gene expression data were derived from RNA sequencing count data, normalized, and log_2_-transformed [log_2_(normalized counts + 1)]. Only tumors with available HER2, ER and PR expression were included.

HER2 sample classification was performed according to the 2023 ASCO/CAP guidelines based on IHC scores and ISH results. Tumors with IHC 3+ or IHC 2+ with ISH positive were classified as HER2-positive, tumors with IHC 1+ or IHC 2+ with ISH negative were classified as HER2-low and tumors with IHC 0 were classified as HER2-negative. HER2-low samples were further subdivided into HER2-low/HR-positive and HER2-low/HR-negative based on HR status.

HR status was classified according to the 2020 ASCO/CAP guidelines for ER and PR testing. Accordingly, HR status was considered positive when ER and/or PR expression were positive. HR status was considered negative when ER and PR expression were negative.

### 2.2. Identification of Deregulated MicroRNAs and Genes

The identification of deregulated miRNAs and genes was performed using the Wilcoxon rank-sum test and DESeq2 R package. Specifically, the Wilcoxon rank-sum test was used for comparisons between TP and NAT samples, as well as between HER2-low/HR-positive and HER2-low/HR-negative samples. Significantly deregulated miRNAs and genes were visualized using the ggplot2 and pheatmap packages. Venn diagrams were produced using the ggvenn package to visualize overlapping significantly deregulated miRNAs and genes across comparisons.

To increase robustness, a differential gene expression analysis was additionally performed on raw gene expression data, comparing HER2-low/HR-positive and HER2-low/HR-negative samples using the DESeq2 R package. Absolute log_2_FoldChange > 1 (|log_2_FoldChange| > 1) and False Discovery Rate (FDR)-adjusted *p*-value < 0.05 were used as thresholds to identify significantly differentially expressed genes (DEGs).

### 2.3. Survival Analysis

Survival analyses were performed separately for HER2-low/HR-positive and HER2-low/HR-negative samples. The overall survival (OS) and progression-free interval (PFI) were evaluated using Cox proportional hazard models implemented via the finalfit R package. For the individual miRNA analysis, expression levels of miRNAs were dichotomized into high and low groups using the median within each HR subgroup, and univariable and multivariable hazard ratios (HRs) were estimated. To assess the combined effect of the selected miRNAs, a composite miRNA signature was generated by standardizing each miRNA expression (z-score), summing the scaled values to obtain a signature score, and dichotomizing into high and low groups based on the median within each HR subgroup. Two-sided *p*-values < 0.05 were considered statistically significant.

### 2.4. Prediction of Target Genes of Deregulated MicroRNAs

Gene identifiers were converted to their corresponding RefSeq mRNA IDs using g:Profiler “https://biit.cs.ut.ee/gprofiler/convert (accessed on November 2025)”. MiRNAs target prediction data were obtained from the miRDB v6.0 “https://mirdb.org/download.html (accessed on December 2025)” and TargetScan v8.0 databases “https://www.targetscan.org/vert_80/ (acecessed on February 2026)” to identify putative downstream target genes of the significantly deregulated miRNAs. Predicted target genes were then intersected with genes showing significant negative correlation.

### 2.5. Functional Analysis

Gene set enrichment analysis (GSEA) was performed to identify biological processes and pathways associated with DEGs. Gene ontology (GO) functional analysis was performed using the gseGO function from the clusterProfiler R package, considering all ontologies: biological process (BP), molecular functional (MF) and cellular component (CC). The significant results were visualized using dot plots and enrichment maps. Kyoto Encyclopedia of Genes and Genomes (KEGG) pathway analysis was performed using the gseKEGG function and significant pathways were visualized with dot plots and further explored using the pathview R package. Furthermore, Reactome pathway enrichment was analyzed using the gsePathway function and significant results were visualized as dot plots and network graphs to show the molecular interactions between enriched Reactome terms.

### 2.6. Statistical Analysis

**Figure 1:**
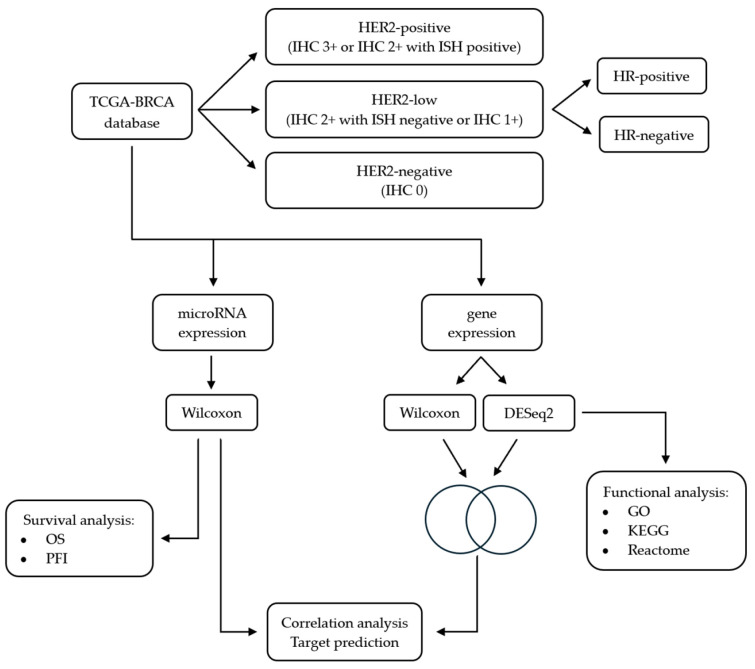
Overview of the study workflow. Breast cancer samples were obtained from the TCGA-BRCA database and classified as HER2-positive, HER2-low and HER2-negative based on IHC scores and ISH results. HER2-low samples were further stratified based on HR status into HER2-low/HR-positive and HER2-low/HR-negative. MiRNA and gene expression data were extracted from the same database. Significantly deregulated miRNAs between HER2-low/HR-positive and HER2-low/HR-negative tumors were identified using the Wilcoxon rank-sum test and subsequently analyzed across all HER2 breast cancer subgroups. The statistically significant miRNAs were evaluated in survival analysis. Significantly deregulated genes between the two HR subgroups were determined using the Wilcoxon rank-sum test and a complementary DEG analysis was conducted to increase robustness. The resulting deregulated genes from the DEG analysis were subjected to functional enrichments analyses. The subset of genes consistently identified across methods was used for integrative correlation and miRNA target prediction analysis to identify putative miRNA–mRNA regulatory networks.

All statistical analyses were performed using R (version 4.4.1). The normality of data distributions was assessed using the Shapiro–Wilk test. Due to the non-normal distribution of expression data, differential expression analysis between two groups was evaluated using the non-parametric Wilcoxon rank-sum test. *P*-values were adjusted for multiple testing using the Benjamini–Hochberg FDR procedure and FDR-adjusted *p*-values < 0.05 were considered statistically significant. Post hoc power analysis was performed to evaluate the impact of sample size imbalance between HER2-low/HR-positive and HER2-low/HR-negative groups. For miRNAs and genes identified as significantly expressed, effect sizes were calculated using Cohen’s d and statistical power was then estimated using a two-sided comparison of means with the observed effect sizes and group sample sizes. The Spearman correlation was used to evaluate the association between a pair of significantly deregulated miRNAs and genes in HER2-low/HR-positive and HER2-low/HR-negative, using the cor.test function in R. Correlation matrices were generated and statistical significance was determined using *p*-values after adjusting for multiple variables. Only negative correlations with coefficients below −0.5 were considered strong and selected for further analysis. During quality control, one NAT sample clustered with tumor samples in the principal component analysis (PCA) and was considered an outlier (Figure S1). This sample was excluded from downstream analysis. Figure 1 illustrates the study workflow.

## 3. Results

### 3.1. Clinicopathological Profile of the Patient Cohort

From a total of 687 samples of breast cancer patients, 597 were tumor samples and 90 were NAT samples. Based on HER2 IHC scores and ISH results, 126 samples were classified as HER2-positive (IHC 3+ or IHC 2+ and ISH positive), 408 samples as HER2-low (IHC 2+ and ISH negative or IHC 1+) and 63 samples as HER2-negative (IHC 0). The HER2-low group was further stratified by HR status, comprising 327 HER2-low/HR-positive and 71 HER2-low/HR-negative tumor samples (Table 1). Most patients in this cohort were female and the mean age varied across groups, with HER2-positive patients being older (59.58 ± 13.38 years) than HER2-low (58.22 ± 13.29 years) and HER2-negative (56.06 ± 12.77 years) patients.

Most tumors were classified as invasive breast carcinoma of no special type (IBC-NST). This pattern was most prominent in the HER2-positive subgroup, whereas lobular histology was relatively more represented among HER2-low cases.

Tumor staging followed the TNM (Tumor–Node–Metastasis) classification system, in which the T stage reflects tumor size (T1–T4), the N stage indicates the presence of regional of lymph node involvement (N0–N3) and the M stage denotes the presence of distant metastasis (M0–M1) [25,26]. HER2-low tumors were more frequently classified as T2, but overall, there was a higher prevalence of T2 tumors in HER2-negative cases. Nodal involvement was most frequent in HER2-positive tumors, whereas HER2-negative patients had the largest proportion of node-negative tumors (58.7%). Distant metastasis was uncommon overall.

Treatment information indicated that nearly all patients did not receive neoadjuvant therapy (98.2%) and radiation therapy was predominantly used in HER2-low patients.

**Table 1:** Clinicopathological characteristics of patients.

		HER2-Positive N (%)	HER2-Low N (%)	HER2-Negative N (%)	Total
**Age** (mean ± SD)		59.58 ± 13.38	58.22 ± 13.29	56.06 ± 12.77	57.95 ± 13.15
**Gender**	Female	122 (96.8%)	404 (99%)	63 (100%)	589 (98.6%)
	Male	4 (3.2%)	4 (1%)	0 (0%)	8 (1.3%)
**Tumor Characteristics**					
**Histological subtype**	Infiltrating Invasive Breast Carcinoma NST	107 (84.9%)	296 (72.5%)	51 (81%)	454 (76.0%)
	Infiltrating Lobular Carcinoma	16 (12.7%)	78 (19.1%)	7 (11.1%)	101 (16.9%)
	Infiltrating Carcinoma NOS	0 (0%)	1 (0.2%)	0 (0%)	1 (0.2%)
	Medullary Carcinoma	0 (0%)	1 (0.2%)	0 (0%)	1 (0.2%)
	Metaplastic Carcinoma	0 (0%)	1 (0.2%)	0 (0%)	1 (0.2%)
	Mucinous Carcinoma	1 (0.8%)	7 (1.7%)	0 (0%)	8 (1.3%)
	Mixed Histology	0 (0%)	9 (2.2%)	0 (0%)	9 (1.5%)
	Other	2 (1.6%)	15 (3.7%)	5 (7.9%)	22 (3.7%)
**Tumor size (T stage)**	T1	17 (13.5%)	84 (20.6%)	14 (22.2%)	115 (19.3%)
	T2	68 (54.0%)	185 (45.3%)	41 (65.1%)	294 (49.2%)
	T3	8 (6.3%)	30 (7.4%)	6 (9.5%)	44 (7.4%)
	T4	5 (4.0%)	10 (2.4%)	2 (3.2%)	17 (2.8%)
	Inconclusive	28 (22.2%)	99 (24.3%)	0 (0%)	127 (21.3%)
**Nodal status (N stage)**	N0	35 (27.8%)	158 (38.7%)	37 (58.7%)	230 (38.5%)
	N1	41 (32.5%)	87 (21.3%)	18 (28.6%)	146 (24.5%)
	N2	13 (10.3%)	44 (10.8%)	4 (6.3%)	61 (10.2%)
	N3	9 (7.1%)	20 (4.9%)	4 (6.3%)	33 (5.5%)
	Inconclusive	28 (22.2%)	99 (24.3%)	0 (0%)	127 (21.3%)
**Metastasis (M stage)**	M0	96 (76.2%)	304 (74.5%)	62 (98.4%)	462 (77.4%)
	M1	1 (0.8%)	5 (1.2%)	1 (1.6%)	7 (1.2%)
	Inconclusive	29 (23.0%)	99 (24.3%)	0 (0%)	128 (21.4%)
**ER status**	Negative	25 (19.8%)	61 (14.9%)	19 (30.2%)	105 (17.6%)
	Positive	73 (57.9%)	259 (63.5%)	44 (69.8%)	376 (63.0%)
	Inconclusive	28 (22.2%)	88 (21.6%)	0 (0%)	116 (19.4%)
**PR status**	Negative	42 (33.3%)	85 (20.8%)	25 (39.7%)	152 (25.5%)
	Positive	57 (45.2%)	234 (57.4%)	38 (60.3%)	329 (55.1%)
	Inconclusive	27 (21.4%)	89 (21.8%)	0 (0%)	116 (19.4%)
**Treatment Information**					
**Neoadjuvant treatment**	NO	123 (97.6%)	402 (98.5%)	61 (96.8%)	586 (98.2%)
	YES	3 (2.4%)	6 (1.5%)	1 (1.6%)	10 (1.7%)
	Inconclusive	0 (0%)	0 (0%)	1 (1.6%)	1 (0.2%)
**Radiation therapy**	NO	51 (40.5%)	165 (40.4%)	26 (41.3%)	242 (40.5%)
	YES	64 (50.8%)	223 (54.7%)	28 (44.4%)	315 (52.8%)
	Inconclusive	11 (8.7%)	20 (4.9%)	9 (14.3%)	40 (6.7%)

### 3.2. Identification of Deregulated MicroRNAs Associated with Hormone Receptor in HER2-Low Breast Cancer

**Figure 2:**
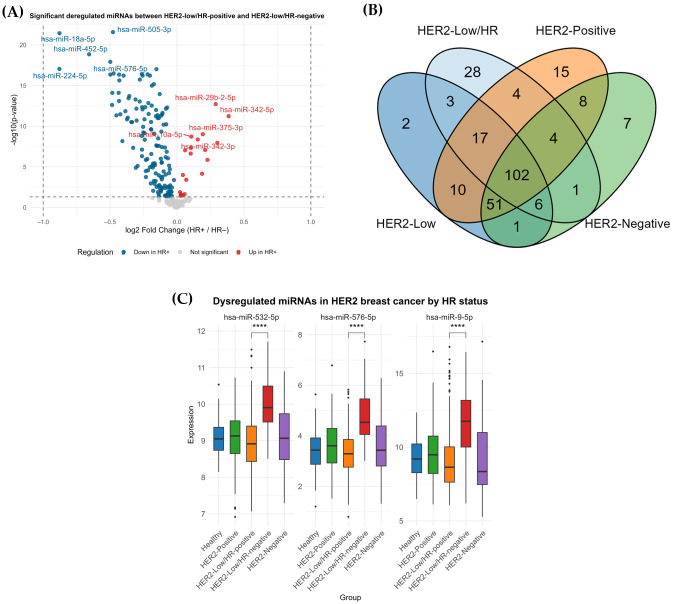
(**A**) Volcano plot of 165 deregulated miRNAs (adjusted *p*-value < 0.05) between 327 HER2-low/HR-positive and 71 HER2-low/HR-negative, of which 145 miRNAs were downregulated (log_2_FC < −1.0) and 20 miRNAs were upregulated in HER2-low/HR-positive (log_2_FC > 1.0). (**B**) All significantly expressed miRNAs obtained from each comparison were intersected in a Venn diagram, which identified 28 miRNAs uniquely deregulated between HER2-low/HR-positive and HER2-low/HR-negative. (**C**) Boxplot demonstrating the miRNA distribution of miR-9-5p, miR-532-5p and miR-576-5p across all HER2 breast cancer subtypes (adjusted *p*-value < 0.05; **** *p* ≤ 0.0001).

Given the central role of miRNAs in the post-transcriptional regulation of gene expression, these molecules may contribute to the heterogenous expression of HR in HER2-low tumors. Comparative analysis of miRNA expressions between TP and NAT samples identified 211 significantly deregulated miRNAs in HER2-positive tumors, 192 significantly deregulated miRNAs in HER2-low tumors, and 180 miRNAs in HER2-negative tumors (Wilcoxon, FDR-adjusted *p*-value < 0.05). Furthermore, differential expression analysis between HER2-low/HR-positive and HER2-low/HR-negative tumors revealed 165 significantly deregulated miRNAs (Figure S2) of which 145 miRNAs were downregulated (log_2_FC < −1.0) and 20 miRNAs were upregulated in HER2-low/HR-positive (log_2_FC > 1.0) (Figure 2A).

To identify miRNAs specifically associated with HR status within HER2-low tumors, a Venn diagram was constructed to intersect the previously obtained miRNA expression datasets (Figure 2B). Results revealed 28 miRNAs uniquely associated with HR status within the HER2-low subtypes. The robustness of these candidate miRNAs was subsequently assessed by comparing the distribution of their expression across all HER2 breast cancer subtypes. Among these, only three miRNAs consistently discriminated HER2-low/HR-positive from HER2-low/HR-negative tumors (Figure 2C). Specifically, hsa-miR-9-5p, hsa-miR-532-5p and hsa-miR-576-5p were consistently upregulated in HER2-low/HR-negative tumors and were, therefore, selected for downstream analyses.

Given the substantial sample size difference between HER2-low/HR-positive (n = 327) and HER2-low/HR-negative (n = 71) groups, post hoc power analysis was performed for these three miRNAs. Results indicated very large effect sizes (Cohen’s d was 1.24, 1.37 and 1.54, respectively) and an estimated power of one for all three, demonstrating that the study was highly powered to detect these differences, despite the smaller size of the HER2-low/HR-negative group.

### 3.3. Prognostic Value of the Three MicroRNAs in HER2-Low/HR-Positive and HER2-Low/HR-Negative Breast Cancer

The prognostic potential of the three miRNAs was evaluated in HER2-low/HR-positive (Tables S1 and S2) and HER2-low/HR-negative groups (Tables S3 and S4). In HER2-low/HR-positive, low expression of miR-9-5p was associated with a non-significant trend toward improved OS (HRs = 0.56, 95% CI 0.23–1.36) and prolonged PFI (HRs = 0.68, 95% CI 0.31–1.51). Low miR-532-5p expression exhibited a minimal effect on OS (HRs = 1.20, 95% CI 0.49–2.94) and PFI (HRs = 1.20, 95% CI 0.52–2.77). In contrast, low expression of miR-576-5p demonstrated a trend toward increased risk of disease progression, particularly for PFI (HRs = 2.06, 95% CI 0.88–4.86). In HER2-low/HR-negative, none of the three miRNAs were significantly associated with OS or PFI, with HRs approximating unity for all comparisons (OS HRs: 0.93–2.41; PFI HRs: 0.90–0.91), likely reflecting the small sample size.

The prognostic value of the miRNA signature was also evaluated In these subgroups (Tables S5 and S6). In the HER2-low/HR-positive group, a low signature was associated with non-significant increase in risk for OS (HRs = 1.28, 95% CI 0.54–3.03) and PFI (HRs = 1.25, 95% CI 0.57–2.76), suggesting a trend toward worse outcomes relative to the high signature group. Conversely, in HER2-low/HR-negative tumors, a low signature exhibited a non-significant trend toward reduced risk for both OS (HRs = 0.38, 95% CI 0.10–1.39) and PFI (HRs = 0.38, 95% CI 0.10–1.39), indicating a possible protective effect. Overall, although neither the individual miRNAs nor the signature demonstrated statistically significant associations with survival outcomes, the divergent trends observed between HER2-low/HR-positive and HER2-low/HR-negative subgroups indicate a potential subgroup-specific prognostic relevance.

### 3.4. Identification of Dysregulated Genes Associated with Hormone Receptor in HER2-Low Breast Cancer

**Figure 3:**
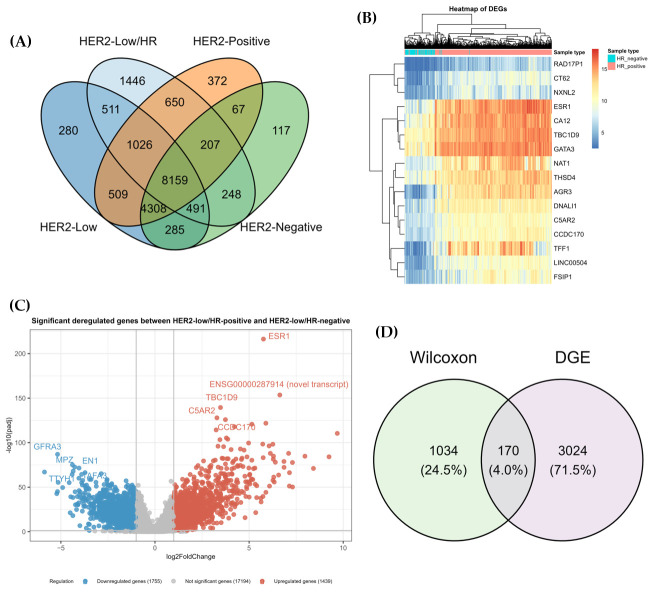
(**A**) All significantly expressed genes obtained from each comparison using the Wilcoxon test were intersected in a Venn diagram, which identified 1446 genes uniquely deregulated between HER2-low/HR-positive and HER2-low/HR-negative. (**B**) Heatmap of the top 20 deregulated genes identified using DEG analysis between 327 HER2-low/HR-positive and 71 HER2-low/HR-negative tumor samples. (**C**) Volcano plot of 3194 deregulated genes using DEG analysis (adjusted *p*-value < 0.05) between 327 HER2-low/HR-positive and 71 HER2-low/HR-negative, of which 1755 genes were downregulated (log_2_FC < −1.0) and 1439 genes were upregulated in HER2-low/HR-positive (log_2_FC > 1.0). (**D**) The identified 1446 significantly deregulated genes using the Wilcoxon test were intersected with the 3194 deregulated genes using a Venn diagram, resulting in 170 common genes.

To identify genes significantly deregulated between study groups, gene expression distributions were compared using the Wilcoxon rank-sum test and were complemented by DEG analysis to enhance the robustness of the results. A total of 15,298 genes were significantly deregulated between HER2-positive tumor and NAT samples, 15,569 genes between HER2-low and NAT samples, and 13,882 genes between HER2-negative and NAT samples (Wilcoxon, FDR-adjusted *p*-value < 0.05). Subsequent comparison of HER2-low/HR-positive and HER2-low/HR-negative tumors identified 12,738 significantly deregulated genes. Intersection analysis using a Venn diagram identified 1446 genes uniquely associated with HR status within the HER2-low subgroup (Figure 3A). Examination of the expression patterns of these 1446 genes across all HER2 subtypes highlighted 171 genes with distinct expression profiles capable of effectively distinguishing HER2-low/HR-positive from HER2-low/HR-negative tumors.

To account for the marked difference in sample size between the HER2-low/HR-positive (n = 327) and HER2-low/HR-negative (n = 71) groups, a post hoc power analysis was conducted for significantly deregulated genes capable of discriminating these two groups. Most genes displayed large to very large effect sizes (|Cohen’s d| ≥ 0.8), reflecting substantial expression differences between the two groups. Correspondingly, these genes showed high estimated statistical power, with values approaching or reaching one. Genes characterized by smaller effect sizes exhibited lower power estimates, consistent with expected statistical behavior.

To further increase the reliability of these results, a complementary DEG analysis was performed to identify the most significantly altered genes between HER2-low/HR-positive and HER2-low/HR-negative tumors (Figure 3B). Of the 3194 deregulated genes identified, 1755 were downregulated (FDR-adjusted *p*-value < 0.05; log_2_FC < −1) and 1439 were upregulated (FDR-adjusted *p*-value < 0.05; log_2_FC > 1) in HER2-low/HR-positive tumors (Figure 3C). Integration of this DEG set with the 1446 uniquely identified in the Wilcoxon analysis revealed 170 overlapping genes, representing a refined subset of consistently deregulated genes across both analytical approaches (Figure 3D).

### 3.5. Functional Analysis of DEGs

**Figure 4:**
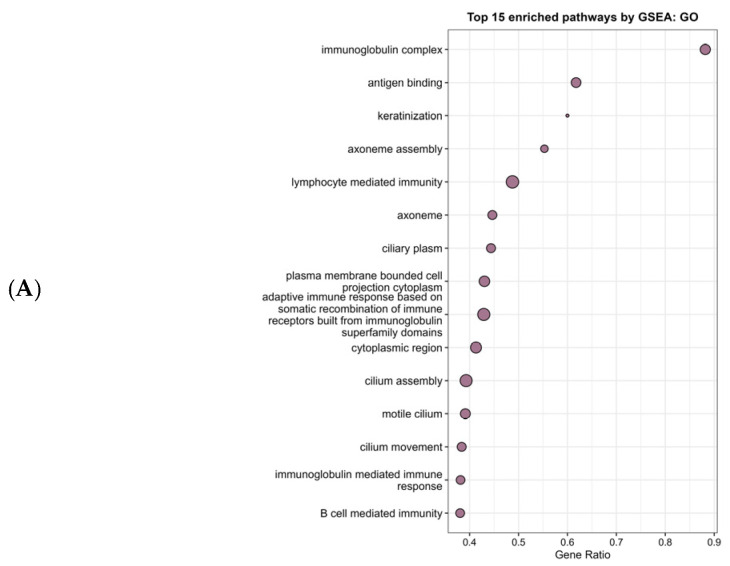

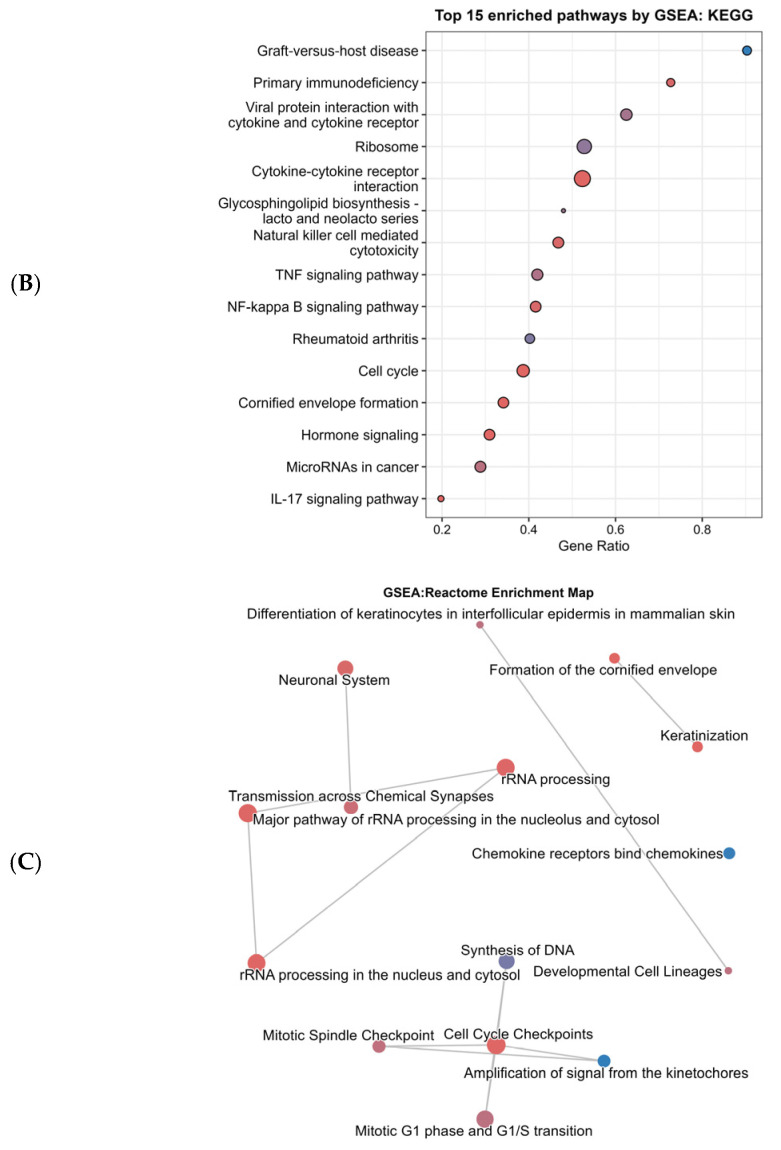
(**A**) Dot plot of the 15 enriched GO pathways. (**B**) Dot plot of the top 15 enriched KEGG pathways. (**C**) Enrichment map of the top 15 enriched Reactome pathways. In the dot plots, dot size represents the number of genes associated with each pathway. In the enrichment map, nodes represent pathways and edges reflect pathway similarity based on shares genes (adjusted *p*-value < 0.05).

To identify biological pathways related to HR expression in HER2-low tumors, we performed GSEA using GO, KEGG and Reactome pathway analyses comparing HR-positive and HR-negative samples. GSEA revealed distinct pathway signatures between both subtypes of tumors. GO functional analysis identified 390 significantly enriched terms (adjusted *p*-value < 0.05), distributed as 60 CC, 37 MF and 293 BP (Figure 4A). HER2-low/HR-positive tumors exhibited a positive enrichment (NES > 0) in pathways related to cilium structure, assembly and motility, including axoneme, ciliary plasm, axoneme assembly, cilium movement and motile cilium. Although not so enriched, hormone-related terms were also identified in these tumors, including the estrogen response element binding, steroid hormone receptor signaling pathway, hormone secretion and regulation of hormone levels. In contrast, HER2-low/HR-negative tumors showed a significant negative enrichment (NES < 0) in pathways relative to humoral and adaptive immune responses, including immunoglobin complex, antigen binding, immunoglobulin-mediated immune response and B cell-mediated immunity. The enriched pathways reveal prominent differences in immune-related and ciliary-associated biological processes between the two groups.

KEGG analysis revealed 8 significantly enriched pathways in HER2-low/HR-positive and 31 significantly enriched pathways in HER2-low/HR-negative, which indicates a strong association with the latter subtype of tumors (Figure 4B). These pathways were predominantly related to immune activation and inflammatory signaling, as pathways such as the IL-17 signaling pathway, NF-κB signaling pathway, cytokine–cytokine receptor interaction and TNF signaling pathway were among the most strongly enriched, suggesting enhanced inflammatory and cytokine-mediated signaling in this tumor subgroup. In addition, multiple pathways related to adaptive and innate immune responses were enriched, including natural killer cell-mediated cytotoxicity and B cell receptor signaling pathway, which further supports the presence of an immune-inflamed transcriptional profile in HER2-low/HR-negative tumors. The smaller subset of pathways positively enriched in HER2-low/HR-positive tumors were hormone-related, including hormone signaling, GnRG secretion, and cortisol synthesis and secretion, which is consistent with HR positivity.

Finally, Reactome pathway analysis was performed to further characterize biological processes differentially enriched between HER2-low/HR-positive and HER2-low/HR-negative (Figure 4C). The most strongly enriched pathways in HER2-low/HR-negative were related to epithelial differentiation and keratinocyte biology, which are indicative of alterations in epithelial differentiation programs. In addition, a large number of negatively enriched pathways were associated with replication stress responses, cell cycle progression, and mitotic control, which support an elevated proliferative and replicative phenotype associated with this tumor subgroup. Consistent with the KEGG analysis, multiple identified pathways were related to immune regulation and cytokine signaling, which suggests a broad activation of the immune responses. By contrast, the positive enriched pathways in HER2-low/HR-positive tumors include estrogen-dependent gene expression, highlighting the presence of hormone-related transcriptional programs.

### 3.6. Correlation Analysis Between Significant MicroRNAs and Genes

**Figure 5:**
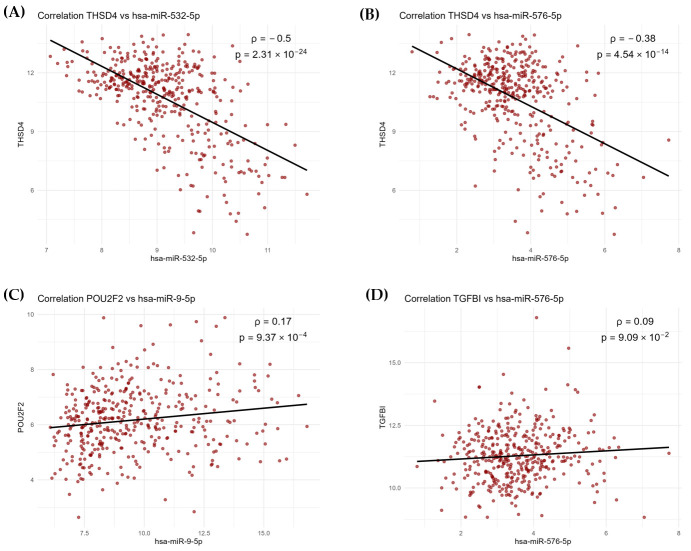
Correlation plots between significantly deregulated miRNAs and genes in HER2-low samples with HR expression. (**A**) Significant negative correlation between THSD4 and miR-532-5p (ρ = −0.5). (**B**) Significant negative correlation between THSD4 and miR-576-5p (ρ = −0.38). (**C**) Significant positive correlation between POU2F2 and miR-9-5p (ρ = 0.17). (**D**) Positive correlation between TGFBI and miR-576-5p (ρ = 0.09), but it did not reach significance.

The potential regulatory relationships between the 170 genes and the three miRNAs were evaluated. Considering the defined cutoff for strong negative correlation (ρ < −0.5; adjusted *p*-value < 0.05), a single significant negative correlation was observed between THSD4 and miR-532-5p, suggesting a potential miRNA–mRNA interaction (Figure 5A). In contrast, target prediction analysis did not suggest THSD4 as a target of miR-532-5p, but instead it was predicted to be regulated by miR-576-5p, with a prediction score of 69.52 in the miRDB database. This interaction was also identified in the correlation analysis (Figure 5B). To further evaluate this interaction, target prediction was additionally performed using the TargetScan database. Although a miRNA–mRNA interaction between THSD4 and miR-576-5p was predicted, it did not meet the predefined criteria for strong targeting (Total context++ score ≤ −0.3 and Aggregate PCT ≥ 0.5), indicating a very weak predicted interaction based on TargetScan scores.

Overall, 52 potential miRNA–mRNA interactions were predicted. Applying a stringent target score cutoff (target score ≥ 80) reduced this set to nine predicted targets (Table S7). Among these, the strongest predicted interactions involved miR-576-5p and TGFBI (target score: 94.70), and miR-9-5p and POU2F2 (target score: 94.17), suggesting particularly robust regulatory relationships. Using the TargetScan database, a total of 59 candidate interactions were predicted. Consistent with the miRDB results, the strongest interactions involved miR-9-5p with POU2F2 and miR-9-5p with TGFBI. Interestingly, POU2F2 was consistently identified as a potential target of miR-9-5p across all three approaches, which may represent a robust candidate for miR-9-5p-mediated regulation. Indeed, the correlation between miR-9-5p and POU2F2 was significant (*p*-value < 0.05) (Figure 5C), whereas the correlation between miR-576-5p and TGFBI did not reach significance (Figure 5D).

## 4. Discussion

HER2-low breast cancer represents an emerging and biologically heterogeneous group of tumors in which HR expression critically influences prognosis and therapeutic decision-making [27,28]. Given the central role of miRNAs in post-transcriptional gene regulation, deregulated miRNA networks may contribute to the modulation of HR status in this subgroup. In this context, our analysis of miRNAs profiles provides new insights into the molecular mechanisms potentially underlying HR heterogeneity in HER2-low breast cancer.

Comparative analysis between TP and NAT samples revealed extensive miRNA deregulation across all HER2 subtypes. The similar magnitude of deregulated miRNAs observed across HER2-positive, HER2-low and HER2-negative tumors underscores the extensive miRNA-driven reprogramming during breast tumorigenesis, regardless of HER2 status. Nevertheless, the specific miRNA signatures differed among subtypes, supporting the existence of distinct regulatory networks defining each HER2 category. Given the biological relevance of HR heterogeneity within HER2-low breast cancer, subsequent analyses focused on identifying miRNAs specifically associated with HR status in this subgroup.

Differential expression analysis between HER2-low/HR-positive and HER2-low/HR-negative tumors identified 165 significantly deregulated miRNAs, suggesting that miRNA regulatory networks may contribute to the divergent HR expression observed within HER2-low tumors. To refine this list and identify miRNAs uniquely associated with HR status in this context, an intersection analysis was performed, yielding 28 candidate miRNAs specifically linked to HR heterogeneity in HER2-low tumors. Further evaluation across all HER2 breast cancer subtypes demonstrated that three miRNAs, namely, miR-9-5p, miR-532-5p and miR-576-5p, consistently distinguished HER2-low/HR-positive from HER2-low/HR-negative tumors. Importantly, all three miRNAs were significantly upregulated in HER2-low/HR-negative tumors, implicating them as potential negative regulators of HR expression.

Consistent with our findings, miR-9-5p has been associated with the luminal-to-basal transition and negatively correlated with ER and PR expression, with ESR1 and PGR reported as direct targets [29]. Additional studies demonstrated that miR-9-5p directly targets androgen receptor (AR) and promotes endocrine resistance via exosome-mediated transfer from tamoxifen-resistant cells [30,31], supporting its role in hormone-independent tumor behavior. Regarding miR-532-5p, prior studies reported its upregulation in breast cancer, particularly in TNBC, where it targets the ras-related and estrogen-regulated growth inhibitor (RERG) and activates MAPK/ERK signaling to promote proliferation and migration [32,33]. This suggests an indirect modulation of HR-related transcriptional programs through oncogenic signaling pathways. Although miR-576-5p remains poorly characterized in the context of breast cancer, its reported downregulation in TNBC and targeting of PIK3CA [34] contrasts with our observation of its upregulation in HER2-low/HR-negative tumors, indicating a context-dependent function potentially influenced by residual HER2 signaling.

Building on these findings, the prognostic relevance of miR-9-5p, miR-532-5p and miR-576-5p was evaluated, both individually and as a composite three-miRNA signature, in HER2-low/HR-positive and HER2-low/HR-negative tumors. Individually, none of the miRNAs demonstrated statistically significant associations with OS or PFI. In HR-positive tumors, low miR-9-5p expression exhibited a non-significant trend toward improved OS and PFI, whereas low miR-576-5p expression was associated with a non-significant trend towards an increased risk of disease progression, particularly for PFI. In HR-negative tumors, HRs for all three miRNAs approximated unity, likely reflecting the limited sample size. Analysis of the composite three-miRNA signature revealed analogous patterns. In HER2-low/HR-positive samples, low signature values were associated with a non-significant increase in risk for OS and PFI, whereas in HER2-low/HR-negative samples, low signature values exhibited a non-significant trend toward reduced risk. Although these associations did not reach statistical significance, the opposing trends observed between the two groups indicate a potential HR-dependent prognostic relevance of these miRNAs that warrants further investigation in larger independent cohorts.

Comprehensive gene expression profiling revealed extensive transcriptional deregulation across all HER2 breast cancer subtypes, highlighting the different molecular landscape associated with breast tumorigenesis. Within HER2-low tumors, comparison between HR-positive and HR-negative cases identified extensive transcriptional differences, indicating that HR status is a major driver of molecular heterogeneity. A refined set of 170 genes retained strong discriminatory power across all HER2-defined groups, showcasing that HER2-low/HR-positive and HER2-low/HR-negative tumors represent biologically distinct entities.

In fact, functional enrichment analysis further highlighted two contrasting biological states. HER2-low/HR-positive tumors were enriched for hormone-related transcriptional programs and epithelial differentiation pathways, consistent with a luminal-like, hormone-dependent phenotype. In contrast, HER2-low/HR-negative tumors displayed enrichment of immune and inflammatory pathways alongside increased proliferative signaling, resembling triple-negative features. Consistent with this, PAM50 analysis of HER2-low/HR-positive tumors have revealed a luminal-like phenotype, enriched with luminal-related gene expression, particularly ESR1, while HER2-low/HR-negative tumors resemble triple-negative features [35,36]. In addition, studies indicate that HR-negative tumors have an increased expression of immune-related genes and thus an enhanced activation of immune pathways alongside pathways associated with increased proliferative activity and altered epithelial differentiation, demonstrated by a higher proliferative index compared to HER2-low/HR-positive and TP53 mutations [35,37]. Also, the presence of an inflamed tumor microenvironment and activation of epithelial–mesenchymal transition (EMT)-related pathways further supports a more aggressive phenotype [38], which is consistent with our data.

Integration of miRNA and mRNA data revealed limited but biologically relevant miRNA–mRNA associations, underscoring the complexity of miRNA-mediated regulation. A strong negative correlation between THSD4 and miR-532-5p supports a potential regulatory relationship. Target prediction analyses further suggested miR-576-5p as a plausible regulator of THSD4, which was also supported by our findings. Although TargetScan predicted an interaction between THSD4 and miR-576-5p, the scores did not meet the predefined criteria for strong targeting, indicating a weak predicted interaction. This gene has recently been linked to extracellular matrix remodeling, immune exclusion and resistance to immunotherapy [39]. This gene is transcriptionally regulated by GATA3 in HR-positive tumors to promote extracellular matrix remodeling, leading cells to acquire a more aggressive phenotype [40]. Among 52 predicted miRNA–mRNA interactions, nine high-confidence interactions [41] were identified, with the miR-9-5p/POU2F2 interaction emerging as particularly robust. Given the involvement of POU2F2 in tumor invasion, immune modulation and resistance to apoptosis [42,43,44], this interaction may contribute to the aggressive phenotype observed in HR-negative tumors.

Although the number of HER2-low/HR-negative tumor samples was smaller than that of HER2-low/HR-positive cases, post hoc power analyses at both miRNA and gene expression levels indicate that this imbalance did not compromise the robustness of the main findings. The three miRNAs that consistently discriminated HR status exhibited very large effect sizes and maximal estimated power, supporting a strong sensitivity to detect true expression differences despite unequal group sizes. Similarly, the majority of genes distinguishing these two groups showed large-to-very large effect sizes accompanied by high power estimates, whereas genes with smaller effect sizes displayed reduced power, as expected. Together, these results suggest that the identified miRNA and gene signatures primarily reflect biologically meaningful differences associated with HR status rather than artifacts driven by sample size imbalance.

From a precision medicine perspective, the identification of miR-9-5p, miR-532-5p and miR-576-5p as discriminative markers between HER2-low/HR-positive and HER2-low/HR-negative tumors highlight their potential utility for refining risk stratification and guiding HR- and immune-targeted therapies. Our integrative analysis demonstrates that HR heterogeneity within HER2-low breast cancer is underpinned by distinct miRNA signatures and transcriptional programs, supporting the existence of biologically divergent tumor states within this emerging subgroup. The consistent identification of a previously unreported miR-9-5p/POU2F2 regulatory axis across integrative analyses highlights a potential mechanism contributing to the aggressive, HR-negative phenotype in this subgroup. Although further functional validation is required, these findings provide a framework for improved molecular stratification of HER2-low breast cancer and may inform the development of more tailored therapeutic strategies.

## 5. Conclusions

In this integrative transcriptomic study of the TCGA-BRCA cohort, we show that HER2-low breast cancer is not a uniform entity but instead comprises biologically divergent tumor states defined by hormone receptor status. HER2-low/HR-positive and HER2-low/HR-negative tumors exhibited distinct miRNA and gene expression landscapes, with three miRNAs (miR-9-5p, miR-532-5p and miR-576-5p) and a refined set of 170 genes emerging as robust discriminators between these subgroups. Functional enrichment analyses consistently highlighted hormone-related and epithelial differentiation programs in HER2-low/HR-positive tumors, in contrast to immune, inflammatory and proliferative signatures predominating in HER2-low/HR-negative disease.

Our findings suggest that specific miRNA–gene networks may contribute to HR heterogeneity and the acquisition of more aggressive phenotypes in HER2-low/HR-negative tumors. In particular, the putative miR-532-5p/THSD4, miR-576-5p/TGFBI and miR-9-5p/POU2F2 regulatory axes link miRNA deregulation to extracellular matrix remodeling, immune modulation and proliferation-associated pathways. From a precision medicine perspective, these miRNAs and their target genes represent candidate biomarkers to refine risk stratification within HER2-low breast cancer and may help inform HR- and immune-directed therapeutic strategies in this emerging subgroup.

This work is based on a retrospective analysis of a single public dataset and lacks functional validation and confirmation in independent cohorts, which should be addressed in future studies. Prospective clinical–translational efforts integrating multi-omics profiling, experimental validation and therapeutic response data will be essential to establish the clinical utility of these miRNA–gene networks as biomarkers and potential therapeutic targets in HER2-low breast cancer.

## Data Availability

All data analyses in this study are publicly available.

## Supplementary Materials

The following supporting information can be downloaded at: https://www.mdpi.com/article/10.3390/genes17030305/s1, Figure S1: PCA of deregulated miRNAs between TP and NAT samples led to the identification of one consistent NAT sample within the tumor cluster. (A) PCA of deregulated miRNAs between HER2-low and NAT samples. (B) PCA of deregulated miRNA between HER2-positive and NAT samples. (C) PCA of deregulated miRNAs between HER2-negative and NAT samples. Figure S2: Boxplot of 165 significantly deregulated miRNAs (*p*-value < 0.05) between 327 HER2-low/HR-positive and 71 HER2-low/HR-negative tumor samples. Table S1: HRs of OS for HER2-low/HR-positive patients for each individual miRNA. CI: confidence interval; HRs: hazard ratios; OS: overall survival. Table S2: HRs of PFI for HER2-low/HR-positive patients for each individual miRNA. CI: confidence interval; HRs: hazard ratios; PFI: progression-free interval. Table S3: HRs of OS for HER2-low/HR-negative patients for each individual miRNA. CI: confidence interval; HRs: hazard ratios; OS: overall survival. Table S4: HRs of PFI for HER2-low/HR-negative patients for each individual miRNA. CI: confidence interval; HRs: hazard ratios; PFI: progression-free interval. Table S5: HRs of OS and PFI for HER2-low/HR-positive patients for miRNA signature. CI: confidence intervale; HRs: hazard ratios; OS: overall survival; PFI: progression-free interval. Table S6: HRs of OS and PFI for HER2-low/HR-negative patients for miRNA signature. CI: confidence intervale; HRs: hazard ratios; OS: overall survival; PFI: progression-free interval. Table S7: MiRNA-mRNA interactions with target prediction score above 80.

## Abbreviations

The following abbreviations are used in this manuscript:

ADC	Antibody–Drug Conjugate
AR	Androgen Receptor
BP	Biological Process
BRCA	Breast Cancer
CC	Cellular Component
DEG	Differently Expressed Gene
EMT	Epithelial–Mesenchymal Transition
ER	Estrogen Receptor
FDR	False Discovery Rate
GO	Gene Ontology
GSEA	Gene Set Enrichment Analysis
HER2	Human Epidermal Growth Factor Receptor 2
HR	Hormone Receptor
HRs	Hazard Ratio
IBC-NST	Invasive Breast Carcinoma of No Special Type
IHC	Immunohistochemistry
ISH	In Situ Hybridization
KEGG	Kyoto Encyclopedia of Genes and Genomes
MF	Molecular Function
miRNAs	MicroRNAs
NAT	Normal Adjacent Tissue
NES	Normalized Enrichment Score
OS	Overall Survival
PCA	Principal Component Analysis
PFI	Progression-Free Interval
PR	Progesterone Receptor
RERG	Ras-related and Estrogen-Regulated Growth Inhibitor
TCGA	The Cancer Genome Atlas
T-DXd	Trastuzumab Deruxtecan
TNBC	Triple-Negative Breast Cancer
TNM	Tumor–Node–Metastasis
TP	Tumor Primary
UTR	Untranslated Region
