# Supplementary material for: MicroRNA–Gene Networks Distinguish Hormone Receptor Status in HER2-Low Breast Cancer: An Integrative Transcriptomic Analysis
Source: Genes (Basel). 2026 Mar 3;17(3):305. doi: 10.3390/genes17030305 (PMC13026672; doi:10.3390/genes17030305)
Supplement: Supplementary file 1 [file genes-17-00305-s001.zip › genes-4121460-supplementary.pdf]

Supplementary Material

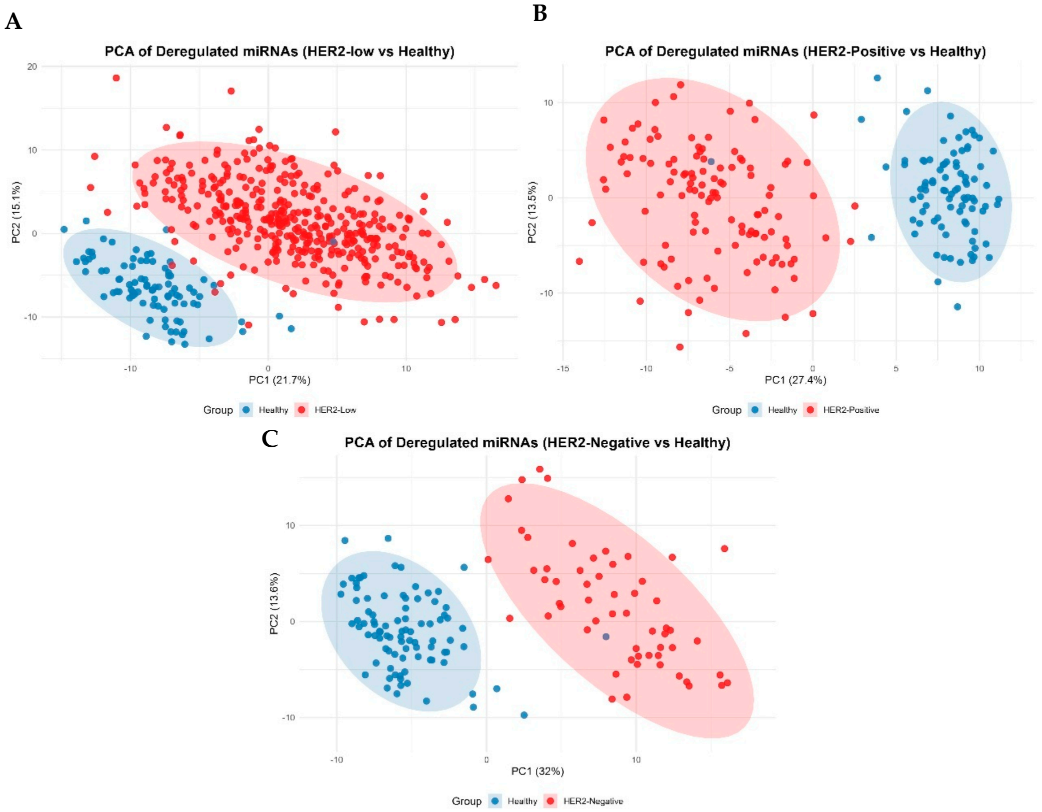

Figure S1: PCA of deregulated miRNAs between TP and NAT samples led to the identification of one consistent NAT sample within the tumor cluster. **A)** PCA of deregulated miRNAs between HER2-low and NAT samples. **B)** PCA of deregulated miRNA between HER2-positive and NAT samples. **C)** PCA of deregulated miRNAs between HER2-negative and NAT samples.

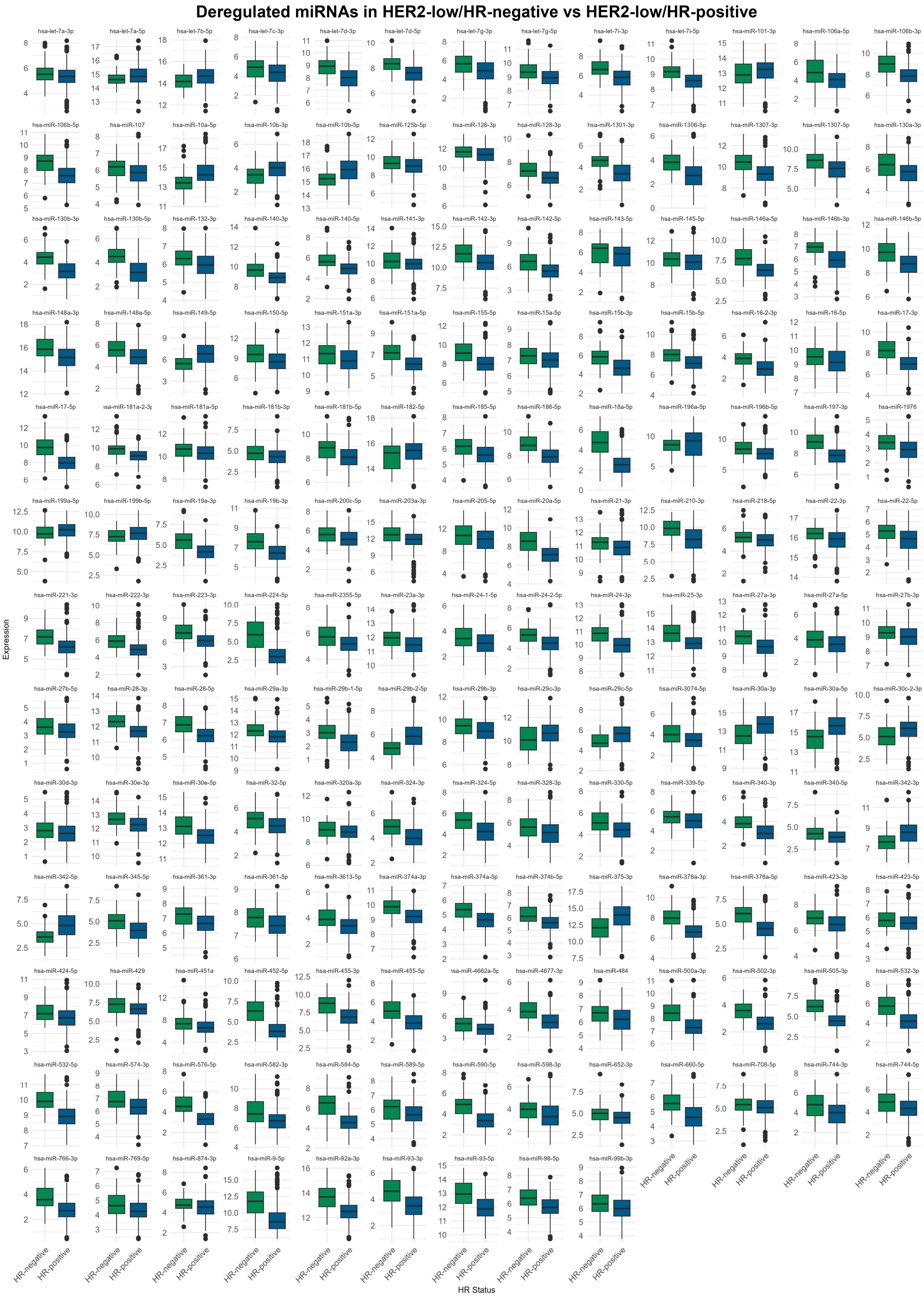

Figure S2: Boxplot of 165 significantly deregulated miRNAs (p-value < 0.05) between 327 HER2-low/HR-positive and 71 HER2-low/HR-negative tumor samples.

Table S1: HRs of OS for HER2-low/HR-positive patients for each individual miRNA. CI: confidence interval; HRs: hazard ratios; OS: overall survival.

			HR (Univariable)		HR (Multivariable)	
**miRNA**	**Level**	**N (%)**	**HR (CI)**	**p-value**	**HR (CI)**	**p-value**
**miR-9-5p**	High	163 (49.8%)				
	Low	164 (50.2%)	0.60 (0.25-1.45)	0.254	0.56 (0.23-1.36)	0.202

**miR-532-5p**	High	163 (49.8%)				
	Low	164 (50.2%)	1.27 (0.54-3.03)	0.582	1.20 (0.49-2.94)	0.696
**miR-576-5p**	High	163 (49.8%)				
	Low	164 (50.2%)	1.50 (0.63-3.59)	0.359	1.54 (0.62-3.79)	0.353

Table S2: HRs of PFI for HER2-low/HR-positive patients for each individual miRNA. CI: confidence interval; HRs: hazard ratios; PFI: progression-free interval.

			**HR (Univariable)**		**HR (Multivariable)**	
**miRNA**	**Level**	**N (%)**	**HR (CI)**	**p-value**	**HR (CI)**	**p-value**
**miR-9-5p**	High	163 (49.8%)				
	Low	164 (50.2%)	0.75 (0.34-1.65)	0.471	0.68 (0.31-1.51)	0.344
**miR-532-5p**	High	163 (49.8%)				
	Low	164 (50.2%)	1.41 (0.63-3.14)	0.399	1.20 (0.52-2.77)	0.667
**miR-576-5p**	High	163 (49.8%)				
	Low	164 (50.2%)	2.09 (0.92-4.75)	0.078	2.06 (0.88-4.86)	0.097

Table S3: HRs of OS for HER2-low/HR-negative patients for each individual miRNA. CI: confidence interval; HRs: hazard ratios; OS: overall survival.

			**HR (Univariable)**		**HR (Multivariable)**	
**miRNA**	**Level**	**N (%)**	**HR (CI)**	**p-value**	**HR (CI)**	**p-value**
**miR-9-5p**	High	35 (49.3%)				
	Low	36 (50.7%)	0.94 (0.25-3.56)	0.925	0.93 (0.23-3.75)	0.919
**miR-532-5p**	High	35 (49.3%)				
	Low	36 (50.7%)	0.95 (0.24-3.81)	0.942	0.69 (0.15-3.13)	0.628
**miR-576-5p**	High	35 (49.3%)				
	Low	36 (50.7%)	2.09 (0.52-8.47)	0.300	2.41 (0.54-10.63)	0.247

Table S4: HRs of PFI for HER2-low/HR-negative patients for each individual miRNA. CI: confidence interval; HRs: hazard ratios; PFI: progression-free interval.

			**HR (Univariable)**		**HR (Multivariable)**	
**miRNA**	**Level**	**N (%)**	**HR (CI)**	**p-value**	**HR (CI)**	**p-value**
**miR-9-5p**	High	35 (49.3%)				
	Low	36 (50.7%)	0.88 (0.29-2.69)	0.821	0.90 (0.29-2.79)	0.851
**miR-532-5p**	High	35 (49.3%)				
	Low	36 (50.7%)	0.84 (0.28-2.50)	0.752	0.89 (0.27-2.94)	0.842
**miR-576-5p**	High	35 (49.3%)				
	Low	36 (50.7%)	0.86 (0.29-2.57)	0.791	0.91 (0.28-2.96)	0.871

Table S5: HRs of OS and PFI for HER2-low/HR-positive patients for miRNA signature. CI: confidence intervale; HRs: hazard ratios; OS: overall survival; PFI: progression-free interval.

			**HR (Univariable)**		**HR (Multivariable)**	

miRNA	Level	N (%)	HR (CI)	p-value	HR (CI)	p-value
**OS**	High	164 (50.2%)				
	Low	163 (49.8%)	1.28 (0.54-3.03)	0.580	1.28 (0.54-3.03)	0.580
**PFI**	High	164 (50.2%)				
	Low	163 (49.8%)	1.25 (0.57-2.76)	0.577	1.25 (0.57-2.76)	0. 577

Table S6: HRs of OS and PFI for HER2-low/HR-negative patients for miRNA signature. CI: confidence intervale; HRs: hazard ratios; OS: overall survival; PFI: progression-free interval.

			HR (Univariable)		HR (Multivariable)	
**miRNA**	**Level**	**N (%)**	**HR (CI)**	**p-value**	**HR (CI)**	**p-value**
**OS**	High	36 (50.7%)				
	Low	35 (49.3%)	0.71 (0.17-3.01)	0.648	0.71 (0.17-3.01)	0.648
**PFI**	High	36 (50.7%)				
	Low	35 (49.3%)	0.38 (0.10-1.39)	0.145	0.38 (0.10-1.39)	0. 145

Table S7: MiRNA-mRNA interactions with target prediction score above 80.

Gene	Transcript	miRNA	Target Score
TGFBI	NM_000358	hsa-miR-9-5p	94.70142
POU2F2	NM_001247994	hsa-miR-9-5p	94.16841
KITLG	NM_003994	hsa-miR-9-5p	86.12571
KITLG	NM_0000899	hsa-miR-9-5p	86.12571
FAM169A	NM_015566	hsa-miR-576-5p	84.75818
PSD3	NM_015310	hsa-miR-9-5p	84.08965
FMN1	NM_001277313	hsa-miR-576-5p	83.16601
FMN1	NM_001103184	hsa-miR-576-5p	83.16601
SYT1	NM_001135805	hsa-miR-9-5p	81.24110
